# COVID-19 in Africa: An Explorative Cross-Sectional Analysis of Twenty-One African Countries From January to June 2020

**DOI:** 10.7759/cureus.24767

**Published:** 2022-05-06

**Authors:** Toluwalase Awoyemi, Ayokunle Adenipekun, Roseline Chima-Kalu, Olubukola Adedayo, Joshua Obarombi, Oluwamayowa Bello, Oluwaseun Bello, Danladi Adamu

**Affiliations:** 1 Nuffield Department of Women's and Reproductive Health, University of Oxford, Oxford, GBR; 2 Emergency Medicine, Health Education England West Midlands, Birmingham, GBR; 3 Pediatrics, University College Hospital, Ibadan, NGA; 4 Internal Medicine, State Hospital, Ijebu-Ode, NGA; 5 Internal Medicine, University College Hospital, Ibadan, NGA; 6 Emergency Medicine, University College Hospital, Ibadan, NGA; 7 Surgery, University College Hospital, Ibadan, NGA; 8 Health Management and Informatics, University of Missouri, Columbia, USA

**Keywords:** covid-19 prevention, sars-cov-2, pandemic, africa, covid-19

## Abstract

Introduction: Africa has surprisingly recorded better gains in containing the coronavirus spread than countries with the better health indices, such as the USA and UK. The low rate of coronavirus disease 2019 (COVID-19) cases and death in Africa represents a puzzle with different biological and social theories such as low COVID-19 testing capacity, substantial young population, few old people, favourable climate, genetic admixture, infectious disease antibodies, and sound community health care systems proposed. We aimed to understand the COVID-19 preventive measures in a group of twenty-one systematically selected African countries that may explain the low burden of COVID-19 in Africa.

Methods: Data (COVID-19, health, socioeconomic, and demographics indices) of twenty-one systemically selected African countries were retrieved from the various official country and multilateral organization sources such as Worldbank, and the United nations development Programme (UNDP). The extracted data were analyzed in three large groups: international travel restrictions, physical and social distancing, and movement restrictions (lockdown measures; curfews, partial or/and national lockdowns). Data cleaning, analysis (including Pearson correlation), and visualization were done with Microsoft Excel and Graph Pad Prism version 9 (https://www.graphpad.com/).

Result: Southern Africa had the greatest number of cases and deaths within the period studied compared to East Africa, which was the least COVID-19 affected sub-region (in terms of COVID-19 cases and deaths). We observed that coronary artery disease death rate was highly correlated with COVID-19 death density (number of COVID-19 deaths/total population) and similarly observed a correlation between the number of cases and deaths and number of in-country arrivals, pandemic preparedness (health security index), COVID-19 containment, and health index (not correlated with deaths). Finally, we noted that the most effective preventive strategy was the 'use of a face mask'.

Conclusion: Africa had fewer COVID-19 cases and COVID-19 related deaths. Our data shows that the rapidity and stringency of COVID-19 preventive measures and government policies, and the low level of tourism in Africa compared to other countries (i.e., low COVID-19 seeding rate) may have been contributory to these favorable statistics. We hope these findings impact how the preparedness for pandemics can be enhanced to decrease the burden of preventable deaths and morbidity.

## Introduction

The novel coronavirus pandemic crippled health systems and economies of countries globally since it was first declared a public health emergency of international concern (PHEIC) on the 30th of January 2020 and then a pandemic on the 11th of March 2020 by the world health organization (WHO) [1-3]. The first case outside China was reported on the 13th of January 2020 in Thailand [4]. Despite the warning, many countries were inadequately prepared and sluggish to establish and enact protective measures to control the virus as the pandemic evolved. Interestingly, in 2019, a year prior to the pandemic, the Johns Hopkins Centre for Health Security and the Nuclear Threat Initiative (NTI), with research by The Economist Intelligence Unit (EIU), released a global health security index that highlighted western countries (the USA, the UK, the Netherlands, and Australia) were more prepared to prevent, detect, and respond to significant disease outbreaks while African countries were the least prepared [5]. This new metric had sparked several debates as it highlighted the weakness of most countries' health security capacities. However, this ranking did not reflect the realities that would happen during the pandemic as the USA and the UK struggled with the pandemic, and lesser ranked countries fared better, predominantly African countries.

The first case in Africa was confirmed in Egypt on the 14th of February 2020 [4]. Several African countries took precautionary measures in line with guidance from WHO and the African center for disease control (Africa CDC) such as travel restrictions, social distancing, lockdowns and use of face masks, curfews, partial or/and national lockdowns, with some even implementing such measures before an index case was reported in the country [6]. The stringency of these measures was relatively high in April 2020, exceeding 65 out of 100 on the Oxford government's response stringency index from the University of Oxford COVID-19 Government response tracker, but had started to dwindle in May 2020 [7]. At the same time, there was global concern about the implication of COVID-19 in Africa and if Africa would be able to handle the pandemic [8,9]. A year after the WHO declared it a PHEIC, more than 100 million cases and 2 million deaths spread worldwide. The USA, India, and Brazil are the worst affected countries. Africa has enjoyed a relatively lower COVID-19 burden when compared to the rest of the world despite poorer healthcare systems, lower literacy levels, higher levels of poverty, and overcrowding relative to Europe or North America. This paradox has been explained by various theories such as higher severe acute respiratory syndrome (SARS-CoV-2) cross-reactive antibodies against, youthful population, favorable weather, effective mitigation measures, low seeding rate due to the low volume of travel to Africa, previous experience with other infectious diseases, regionalism, collaboration and cooperation among countries and the African scientific community [10-13]. We studied the preventive measures, the natural course of the pandemic and pre-pandemic health indices, demographics, and health status of twenty-one African countries to identify factors that may explain the low COVID-19 burden in Africa.

## Materials and methods

Country selection

**Figure 1:**
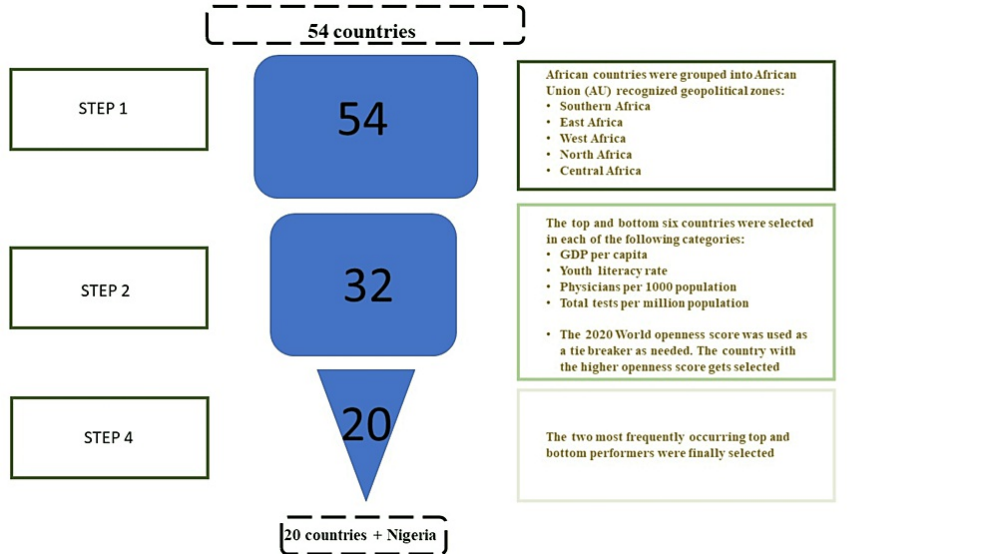
Summary of the country selection methods used in this study There were three steps involved; step 1 involved the classification of the African countries into African Union (AU) recognized geopolitical zones, step 2 involved the selection of the top and bottom six countries using four metrics, and step 3 involved the passage of the two most occurring top and bottom countries in each geopolitical zones. Nigeria was included as the 21st country due to its socio-economic role and population in Africa

Twenty-one African countries were systemically selected using multiple parameters (stated below) to get appropriate representation across several countries. We focused on these twenty-one countries because of the time and resource constraints we would have incurred by studying all fifty-four African countries. The countries were initially grouped according to the African Union recognized geopolitical regions; North, Central, West, East, and Southern Africa (Step 1). The countries in each region were ranked and the top and bottom six performers based were selected based on socioeconomic metrics, level of medical care, literacy level, and COVID-19 testing capabilities (Step 2). Indicators used for these categories were Gross Domestic Product (GDP) Per Capita, youth literacy rate (metrics for economy/purchasing power), Physicians/1000 population and total tests/million population (metrics for the level of medical care, COVID-19 pandemic preparedness). We selected these metrics as part of the screening criteria because we believed they were great proxies for the level of medical care, COVID-19 pandemic preparedness and economy/purchasing power of the countries. In instances of a tie between countries, we selected the country with the highest 2020 world openness score/passport index rank, an index of global mobility (see data source in supplementary data). We chose the 2020 world openness score as a tiebreaker as it acts as a measure of a country's tourism potential and by extension the potential for the spread of an infectious agent, in this case, COVID-19. After identification, of the top and bottom, six performers in each category, the two most frequently occurring top and bottom countries across all criteria and sub-region were selected for inclusion in the study (step 3) (Table 1). Nigeria was included in the study due to its significant influence on other African countries due to its population, trade, and economic contributions, which could play essential parts in the transmission and spread of the pandemic [14-15]. Figure 1 contains a summary of our screening and selection criteria.

 

**Table 1:** Classification of the twenty-one African countries used in this study. * Southern Africa refers to the South Africa sub-region while South Africa refers to the country. **Nigeria's inclusion is discussed in the text

	Southern Africa	East Africa	West Africa	North Africa	Central Africa
Top performers	South Africa	Mauritius	Ghana	Libya	Gabon
	Botswana	Seychelles	Cape Verde	Algeria	Sao Tome and principe
			Nigeria**		
Worst Performers	Malawi	Somalia	Niger	Egypt	Central African Republic
	Mozambique	Tanzania	Benin	Morocco	Burundi

Data collection

Country-specific preventive measure data and COVID-19 statistics from January 22, 2020, to June 30, 2020, were gathered from several official sources, including official government statements, regular gazettes, published guidelines, WHO, Africa CDC guidelines, and COVID-19 tracking websites (the link to the dataset and their sources, and access dates can be seen in the supplementary section). The responses assessed in this study were analyzed in three large groups: International travel restrictions, physical and social distancing, and movement restrictions (lockdown measures; curfews, partial or/and national lockdowns). We also gathered data and metrics on health indicators (hospital beds/10,000 population, life expectancy {2015-2020} and health care security index), socioeconomic indicators (youth/adult literacy rate, age dependency ratio, GDP, median age {2020}), and pre-existing mortality indicators (hypertension death per 100,000, coronary artery disease death per 100,000, human development index, diabetes mellitus death per 100,000, human immunodeficiency virus/acquired immunodeficiency syndrome (HIV/AIDS) death per 100,000, kidney disease death per 100,000 and tuberculosis death per 100,000) (see the supplementary section for the dataset and sources used in this study). We used the earliest and most comprehensive published preventive measure for each country (from the official or/and reliable communication channel(s) of each country). It is important to note that throughout the six months the data for this study was conducted, there were minimal changes in the guidelines. Due to the enormity of the extracted data, we have included and publicly uploaded all relevant information through a link that is accessible in the supplementary data. 

Data analysis

Case density was calculated by dividing the country of interest’s COVID-19 cases by the total population while death density was calculated by dividing the country of interest’s COVID-19 deaths by the total population (obtained using the 2020 United Nations population division estimates). Fold change (at two or three weeks) was calculated by dividing the number of cases or deaths two or three weeks after implementation of a preventive strategy by the number of cases or deaths on the day the preventive strategy was implemented. We selected two data points; two and three weeks. Two weeks because 1) the reported incubation period of SARS-CoV 2 at the time of the study was 2 to 14 days, and 2) because all countries chose the quarantine period to be 14 days [16-17]. We arbitrarily added a second collection point at three weeks (one week extra) to allow for full elucidation of each preventive measure, and to increase our confidence in the result. Data collection, cleaning, Pearson correlation, and visualization (with line graphs, and stacked barplot) were done using Microsoft Excel and Graph Pad Prism version 9 (www.graphpad.com). Pearson correlation was chosen to assess the linear relationship between COVID-19 cases, COVID-19 deaths, COVID-19 case density, and COVID-19 death density and independent variables (health indicators, socioeconomic indicators, and pre-existing mortality indicators) earlier discussed in the methods section. Significance was set at a P-value of < 0.05.

## Results

COVID-19 statistics, population, and previous pandemic experience of the continents

To better understand the COVID-19 story in Africa, we compared Africa to other continents. Asia is the most populated continent with about 4.6 billion people and had the highest number of COVID 19 cases (62, 362, 678 cases). In comparison, Oceania, the least populated (43 million people) had the least COVID-19 cases (106, 276 cases), the least case density ratio (number of cases/population), and the least number of deaths. Europe (51,609,898 people) and North America (42,634, 503 people) had the second and the third highest cases, respectively. Europe had the highest number of total deaths, but South America had the most death and case density. Unlike Europe, North America, and Oceania, which had not previously experienced an epidemic or outbreak in the last five years, Africa recently experienced the Ebola outbreak, Asia had the MERS (Middle Eastern Respiratory Syndrome) outbreak, and South America had the Zika virus outbreak (Table 2).

**Table 2:** Demographics of the seven continents of the world and related COVID-19 statistics, disease outbreaks and population. Data assessed on 8/2/2021

Continent	COVID-19 Cases	COVID-19 Case density	COVID-19 Deaths	COVID-19 Death density	Population	Disease outbreaks in the last five years	Year of Disease outbreaks
Africa	6,802,818	0.01	171,479	<0.01	1,340,598,147	Ebola	2014-2016
Asia	62,362,678	0.01	902,638	<0.01	4,641,054,775	MERS	2015-now
Europe	51,609,898	0.07	1,134,982	<0.01	747,636,026	Nil	Nil
South America	35,579,351	0.08	1,091,089	<0.01	430,759,766	Zika	2015-2016
Oceania	106,276	0.00	1,541	<0.01	43,111,704	Nil	Nil
North America	42,634,503	0.07	941,198	<0.01	592,072,212	Nil	Nil

Age dependency ratio and the youth-adult literacy rate of the study population

Mauritius had the highest age dependency ratio and the highest proportion of the population above 65 years, while Burundi had the lowest age dependency ratio (Table 3). The Central Africa sub-region had the lowest average age dependency and proportion of population aged above 65 years of all the regions (Table 3). East African nations had the highest proportion of population aged above 65 years. Nigeria and Niger, respectively, had the least age dependency ratio and population above 65 years in West Africa (Table 3). Finally, East Africans were the most literate (average youth literacy rate = 94.62), while west Africans were the least literate (average youth literacy rate = 74.01). Seychelles was the most literate country (average youth literacy rate= 99.07) and the Central African Republic the least literate (average youth literacy rate =38.27).

**Table 3:** Pre-COVID-19 health and sociodemographic data of the countries in this study * To prevent confusion, Southern Africa was used to denote the South Africa sub-region while South Africa was used to represent the country. N/A: Not available

Country	Youth/Adult Literacy rate	Total tests/million	Age Dependency ratio (old)	Hospital Beds/10 000 population	Life expectancy (total)	Populations Aged 65 and above	COVID-19 containment and health index	Health security index
NORTH AFRICA								
Algeria	97.43	5,164	10.79	19.00	76.88	6.74	32.24	23.60
Libya	N/A	188,615	6.69	32.00	72.91	4.53	39.69	25.70
Egypt	88.19	29,388	8.78	14.30	71.99	5.33	38.32	39.90
Morocco	97.73	204,486	11.59	10.00	76.68	7.60	42.84	43.70
Average	94.45	106,913	9.46	18.83	74.62	6.05	38.27	33.23
WEST AFRICA								
Benin	60.95	48,479	5.99	5.00	61.77	3.28	31.60	28.80
Cape Verde	98.11	354,684	7.13	21.00	72.98	4.79	36.26	29.30
Nigeria	75.03	11,528	5.09	5.00	54.69	2.74	41.75	37.80
Ghana	92.49	44,596	5.26	9.00	64.07	3.14	33.05	35.50
Niger	43.46	5,172	5.44	3.90	62.42	2.53	23.75	32.20
Average	74.008	92,891.8	5.78	8.78	63.19	3.29	33.28	32.72
CENTRAL AFRICA								
Central African Republic	38.27	11,140	5.21	10.00	53.28	2.80	23.76	27.30
Burundi	88.22	28,173	4.55	7.90	61.58	2.38	10.10	22.80
Sao Tome and Principe	97.78	63,833	5.45	29.00	70.39	3.01	N/A	17.70
Gabon	89.78	429,918	5.96	13.00	66.47	3.53	38.99	20.00
Average	78.5125	133,266	5.29	14.98	62.93	2.93	24.28	21.95
EAST AFRICA								
Mauritius	99.04	281,523	17.71	34.00	74.24	12.52	33.58	34.90
Tanzania	85.76	N/A	4.91	7.00	65.46	2.64	19.79	36.40
Seychelles	99.07	217,199	11.83	36.00	73.94	8.07	37.66	31.90
Somalia	N/A	10,330	5.70	8.70	57.40	2.90	23.25	16.60
Average	94.62	169,684	10.04	21.43	67.76	6.53	28.57	29.95
SOUTHERN AFRICA*								
South Africa	95.32	247,868	8.39	23.00	64.13	5.51	42.66	54.80
Mozambique	70.91	23,077	5.39	7.00	60.85	2.86	26.17	28.10
Botswana	N/A	612,147	7.27	18.00	69.59	4.51	34.78	31.10
Malawi	72.94	17,308	4.86	13.00	64.26	2.64	25.12	28.00
Average	79.72	225,100	6.48	15.25	64.71	3.88	32.18	35.50

Pre-COVID-19 health indices of the study population

North African countries had the highest number of physicians per thousand, the highest COVID-19 containment and health indices per region, and the highest male, female, and total life expectancies. East African countries had the highest number of hospital beds per ten thousand (shown in Table 3). Southern African countries had the best health security index, a measure of pandemic preparedness (shown in Table 3). West African countries had the least female life expectancy, the least number of physicians per thousand, and hospital beds per ten thousand. Finally, Central African countries had the worst health security indices, and COVID-19 containment and health indices per region, the lowest male and total life expectancies (shown in Table 3), and the highest Maternal Mortality Ratio average. In summary, North, South, and East Africa had better health (pre-COVID-19 and COVID-19) indices than West and Central Africa.

COVID-19 cases, deaths, and test rates in Africa

**Figure 2:**
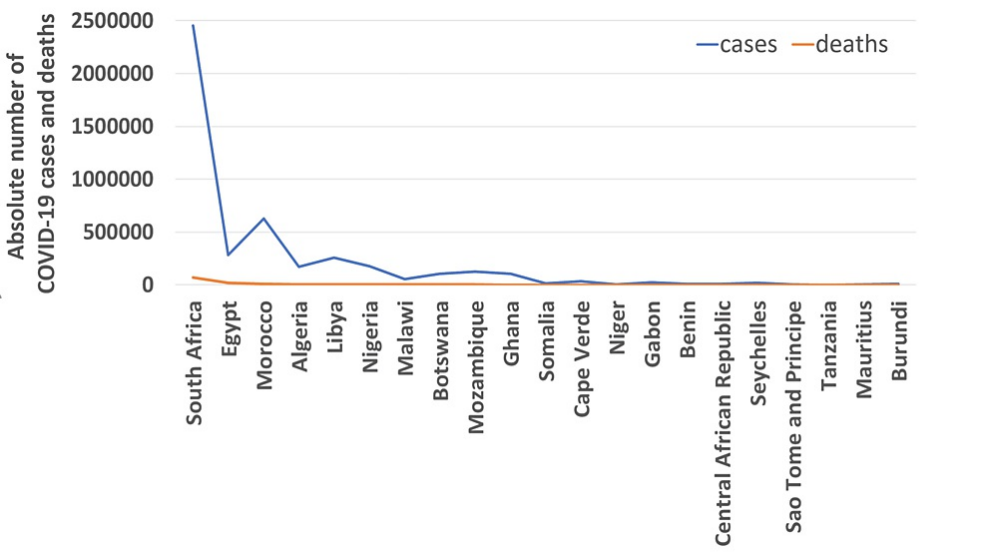
Line graph of COVID-19 case and death of the twenty-one African countries studied. The Y-axis shows the absolute number of cases over the period of study (January 22, 2020, to June 30, 2020) while the X-axis shows the twenty-one African countries included in this study.

Among the countries studied, South Africa (Figure 2) had the highest number of cases (2,456,184 cases) and deaths (72,191 deaths) in contrast with 1,017 cases reported in Tanzania - the least number of cases and Burundi-the least number of deaths (nine deaths). Likewise, the Southern Africa sub-region reported the highest number of cases and deaths while East Africa reported the least number of cases and Central Africa reported the least deaths. Interestingly, Botswana carried out the highest number of tests for COVID-19 (612,147 tests per million) and Algeria tested the least (5164 tests per million) (shown in Table 3). As expected, Southern Africa had the highest testing capacity (225,100 per million tests per million), but West Africa (92,2891.8 tests per million) had the least testing capacity.

Percent of inbound tourism (flights) from United Nations World Tourism Organization (UNWTO) regions to African countries before the COVID-19 pandemic

**Figure 3:**
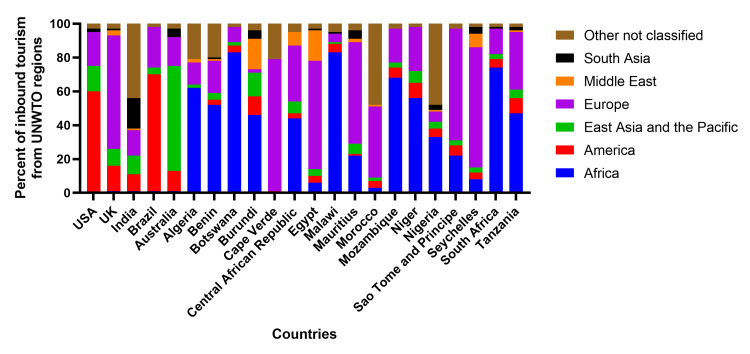
Stacked bar plots showing the inbound tourism (flights) from UNWTO regions to African countries before the COVID-19 pandemic. The Y-axis shows the percent of inbound tourism (flights) from United Nations World Tourism Organization (UNWTO) regions to seventeen of the twenty-one African countries and the United States of America (USA), United Kingdom (UK), India, Brazil, and Australia. We found no inbound tourism data for four countries (Libya, Ghana, Gabon, and Somalia). The United States of America (USA), United Kingdom (UK), India, and Brazil, were included in this stacked bar plot because they had the highest rates of COVID-19 cases and deaths at various points during the period of study and manuscript writing while Australia was included due to its relatively low COVID-19 cases and death toll.

On average, 41% of inbound flights to African countries in our study were from within Africa, Europe accounted for 33%, and East Asia, Southeast Asia, and the Pacific accounted for 5% (Figure 3). Compared to the USA, most of the inbound flights (60%) were from America, while Europe accounted for 20%. Southeast Asia and the Pacific accounted for 17% of these flights (Figure 3). Similarly, the majority of U.K inbound flights were from Europe (67%). America accounted for 15%, and Southeast Asia and the Pacific accounted for 11% of the flights (Figure 2). Brazil had most of its arrivals (69%) from America, while Europe accounted for 24% and Southeast Asia and the Pacific accounted for 4% of the flights (Figure 3). Interestingly, most of the arrivals to India were unclassified (44%), 15% from Europe, 29% from Southeast Asia, and the Pacific accounted for 4% of the flights. Finally, Australia had 67% of its arrivals from Southeast Asia and the Pacific, 12% from America, and 17% from Europe (Figure 3).

Relationship between COVID-19 and GDP of the countries studied

**Figure 4:**
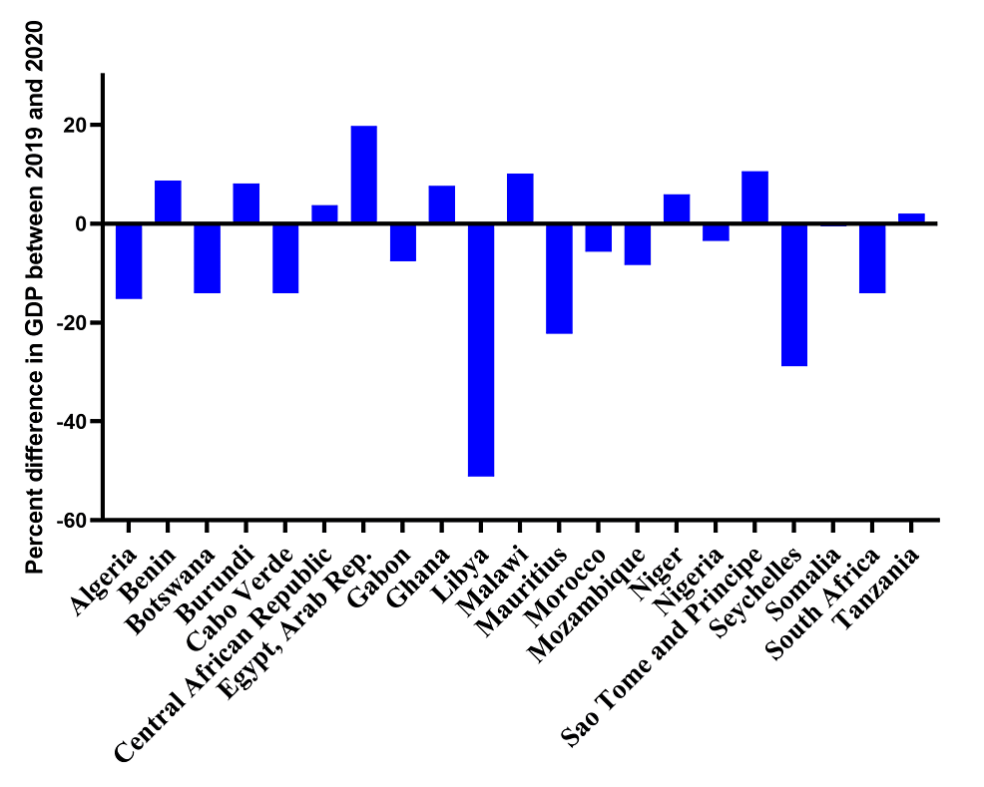
The impact of the COVID-19 pandemic on the gross domestic product among the twenty-one African countries studied in this project. The Y-axis shows the difference in the gross domestic product (GDP) between 2019 (pre-pandemic) and 2020 ( during the pandemic) while the X-axis shows the twenty-one African countries in this study.

There was a change in GDP in 2020 compared to 2019, with about fifty-seven percent (12 out of 21) of countries studied showing a decline whilst others showed a minimal increase (Figure 4). The Southern African region recorded the most significant reduction (mean drop of 13.06 billion USD). South Africa had the single most significant drop (nearly 50 billion USD), making it the most affected of all the countries studied. The second and third most impacted countries are Libya (a 26.67 billion USD reduction) and Algeria (a 25.99 billion USD reduction) respectively, both in the North African region. Nine countries in our study experienced again in GDP from 2019 to 2020, with Egypt recording the highest increase, an approximate 60 billion USD gain. Overall, all regions experienced an average reduction, excluding North Africa, which recorded an average gain of 0.12 billion USD.

Relationship between prevention strategies and COVID-19 cases at two and three weeks after implementation

**Figure 5:**
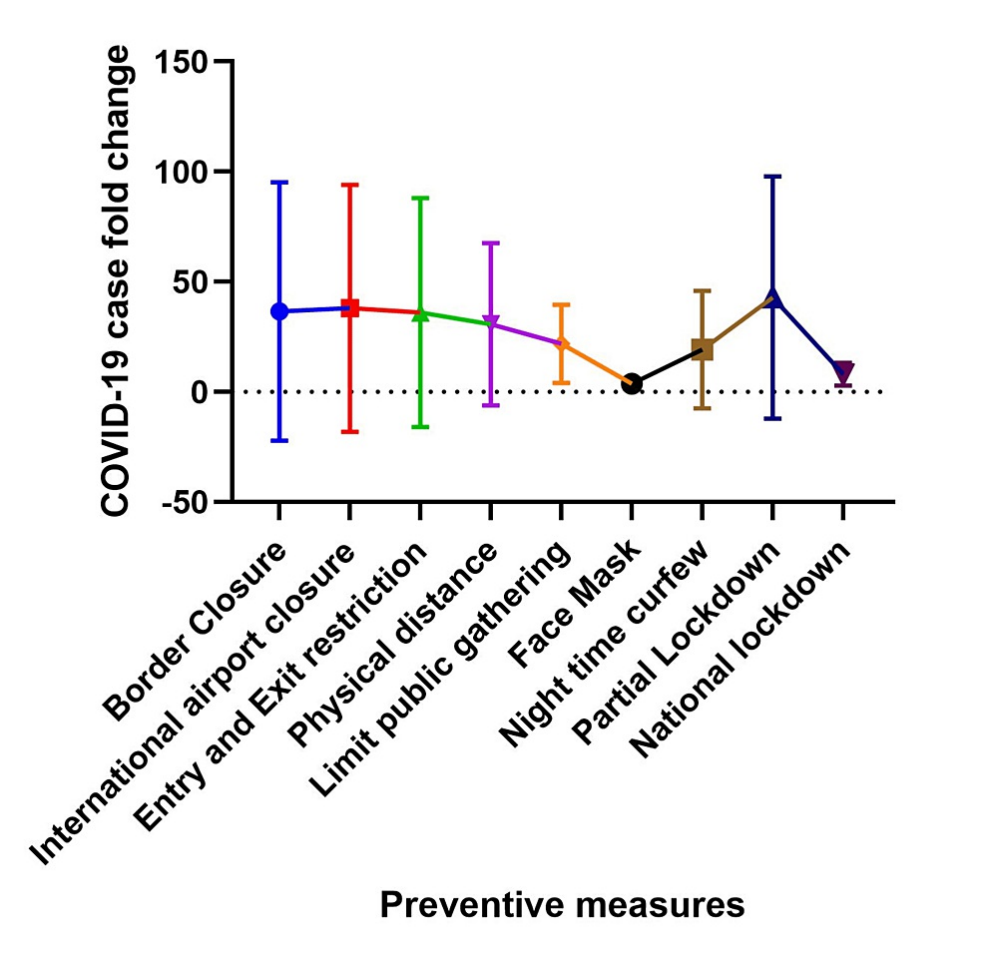
Relationship between COVID-19 preventive measures and COVID-19 caseload two weeks after implementation. The Y-axis shows the COVID-19 case fold change, which measures the change in COVID-19 cases two weeks after a preventive measure was announced compared to the number of cases on the day the preventive measure was implemented. The X-axis shows the COVID-19 preventive measures.

**Figure 6:**
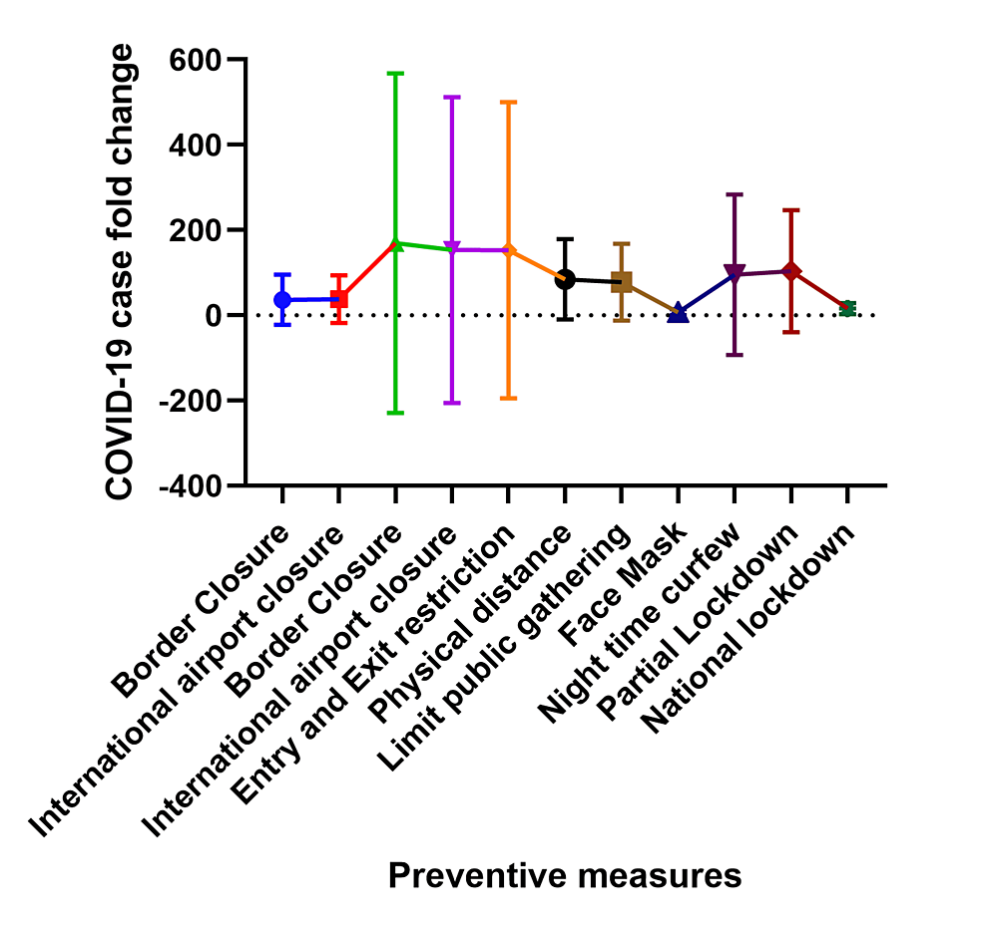
Relationship between COVID-19 preventive measures and COVID-19 caseload three weeks after implementation. The Y-axis shows the COVID-19 case fold change, which measures the change in COVID-19 cases three weeks after a preventive measure was announced compared to the number of cases on the day the preventive measure was implemented. The X-axis shows the COVID-19 preventive measures.

This relationship between prevention strategies and COVID-19 cases covered in this section is best interpreted as a blunting or flattening of the fold change rather than an increase in fold change, for example, the prevention measures work to reduce the magnitude of the fold change and thus the most effective prevention methods have the least fold change and vice versa. Figures 5 and 6 show the fold change in cases at two weeks and three weeks after instituting an intervention in each country. The number of cases after border closure increased as much as 193 folds (at two weeks) and between 6 and 1612 folds (at three weeks) post-implementation. After international airport closure, the number of cases increased as much as 193 folds two weeks after and 3 to 1612 folds three weeks after closing international airports. Entry and exit restrictions corresponded with an increase in the number of cases as much as 193 folds two weeks after and as much as 1612 folds three weeks after.

After the institution of physical/social distance measures, the number of cases increased as much as 167 (at two weeks) and 3 to 427 folds (at three weeks). Likewise, when public gatherings were limited, there was an increase between 3 to 56 folds (two weeks) and 4 to 411 folds (at three weeks) in the number of cases. Promoting the use of face masks resulted in a 2 to 10 folds (at two weeks) and a 2 to 29 folds (at three weeks) increase in cases. Commencing night-time curfew corresponded with an increase in cases by 2 to 52 folds (at two weeks) and 3 to 595 folds (three weeks). Despite commencing partial lockdown, the number of cases increased by 4 to 167 folds (two weeks) and 8 to 428 folds (three weeks), while with national lockdown, the number of cases increased by 2 to 15 folds (at two weeks) and 2 to 38 folds (at three weeks). In general, face masks and national lockdown were the most effective preventive measures for the number of cases two and three weeks post-intervention.

Relationship between prevention strategies and COVID-19 deaths at two and three weeks after implementation

**Figure 7:**
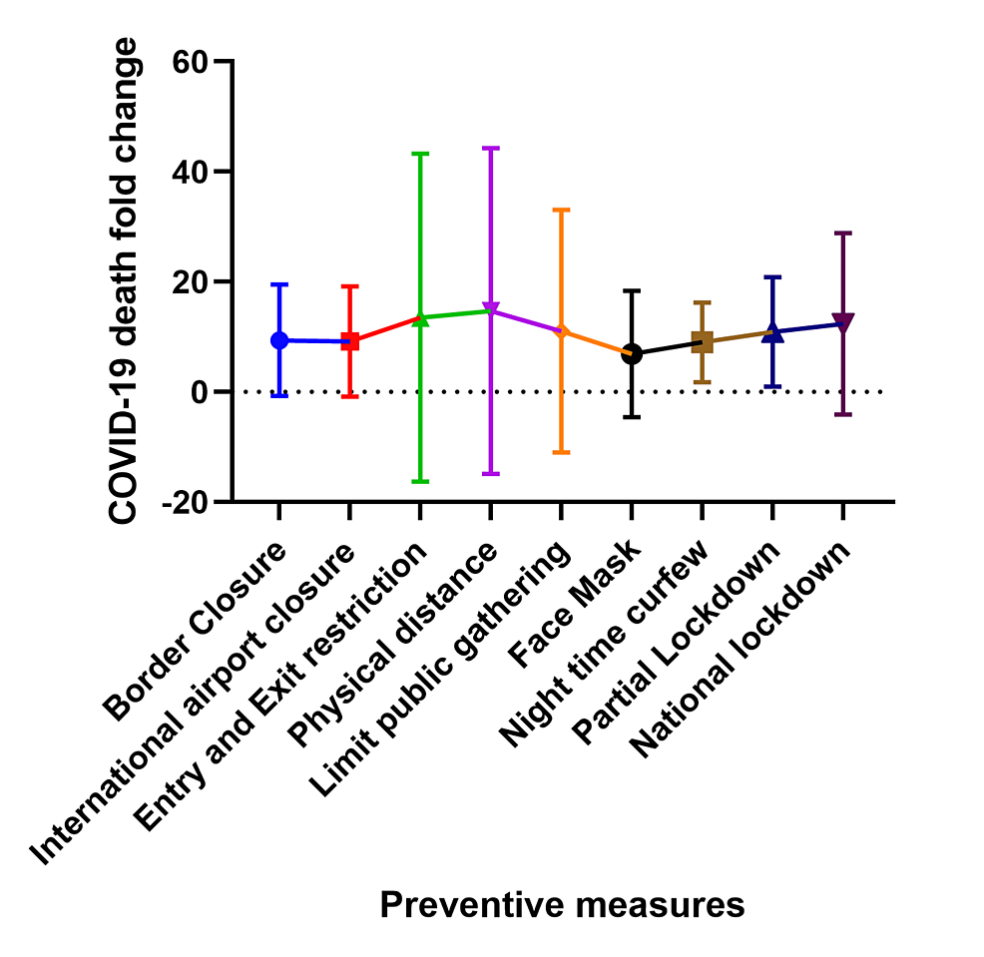
Relationship between COVID-19 preventive measures and COVID-19 death fold change two weeks after implementation. The Y-axis shows the fold change which is a measure of the difference in COVID-19 death two weeks after a preventive measure was announced compared to the number of deaths on the day the preventive measure was implemented. The X-axis shows the COVID-19 preventive measures.

**Figure 8:**
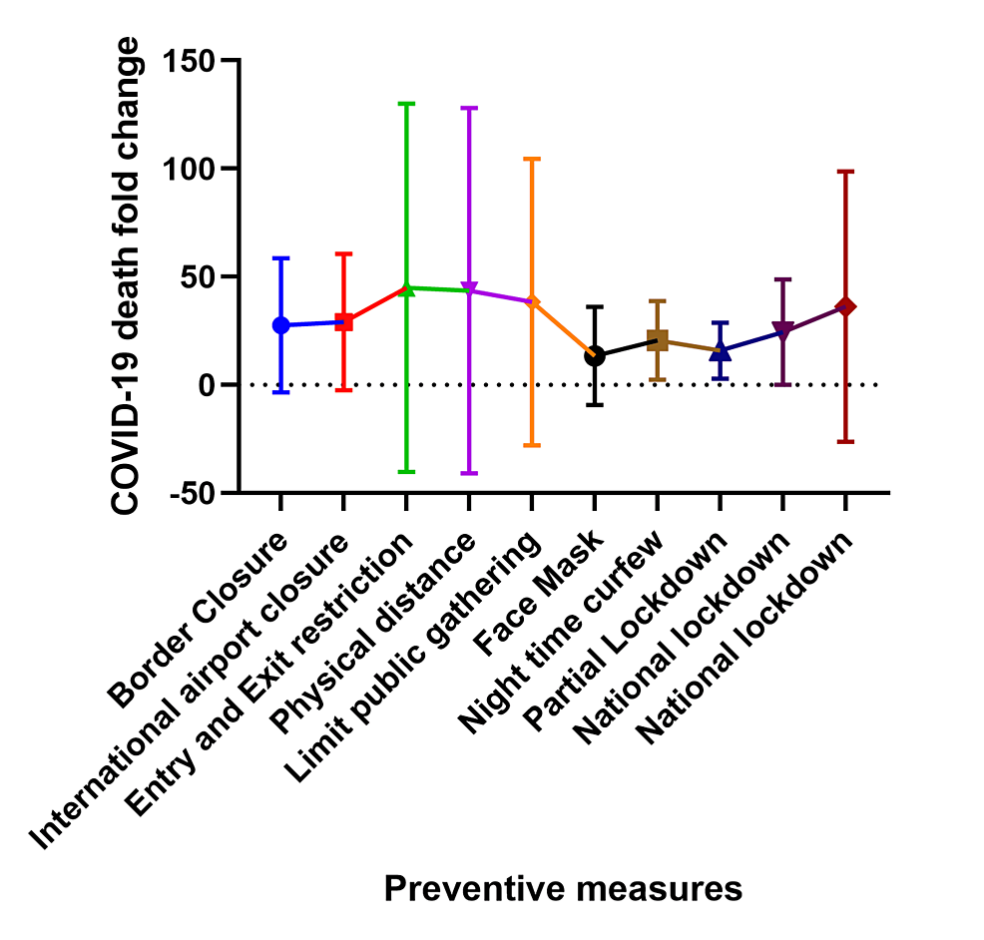
Relationship between COVID-19 preventive measures and COVID-19 death fold change three weeks after implementation. The Y-axis shows the fold change which is a measure of the difference in COVID-19 death three weeks after a preventive measure was announced compared to the number of deaths on the day the preventive measure was implemented. The X-axis shows the COVID-19 preventive measures.

This relationship between prevention strategies and COVID-19 deaths covered in this section is best interpreted as a blunting or flattening of the fold change rather than an increase in fold change, for example, the prevention measures work to reduce the magnitude of the fold change and thus the most effective prevention methods have the least fold change and vice versa. Figures 7 and 8 show the fold increase in cases at two weeks and three weeks following preventive measures in each country. Following border and international airport closure, the number of deaths increased as much as 32 folds (at two weeks) and 81 folds (at three weeks) after implementation. After commencing physical/social distancing, entry, and exit restriction, there was an increase in the number of deaths up to 134 folds (at two weeks) and 382 folds (at three weeks). Similarly, when public gatherings were limited, the number of deaths increased as much as 94 folds (at two weeks) to 280 folds (at three weeks). When the use of face masks was recommended, the number of deaths increased as much as 39 folds (at two weeks) and 64 folds (at three weeks) after the intervention, while the number of deaths increased by 3 to 25 folds (at two weeks) and 4 to 48 folds ( at three weeks) after night-time curfew.

Following a partial lockdown, the deaths increased by 2 to 32 folds (at two weeks) and 3 to 81 folds (at three weeks) after implementation. Similarly, with national lockdown, deaths increased as much as 51 folds (at two weeks) and 186 folds (at three weeks) after implementation. In general, face masks (at two and three weeks) and partial lockdown (at two weeks) had the best preventive effect on the number of deaths post-implementation.

Correlation between preventive measures, health, and development metrics and COVID-19 cases and deaths

We performed correlation analysis between total cases, case density, total deaths, and death density and socioeconomic indicators (the number of arrivals, populations aged 65 and above, population, median age, human development index, and life expectancy), health indicators (COVID-19 containment and health index, hospital beds/10 000 population, total COVID-19 tests per million and health security index) and pre-existing mortality indicators (hypertension, coronary heart disease, diabetes mellitus, human immunodeficiency virus/acquired immunodeficiency syndrome (HIV/AIDS), kidney disease and tuberculosis death rate per 100,000). There was a statistically significant correlation between total number of cases with total deaths (r=0.87, p= <0.0001) number of arrivals into the country (r=0.78, p=<0.0001), health security index (r=0.72, p= 0.0003), COVID-19 containment and health index (r=0.46 p=0.04). Similarly, total deaths were significantly correlated with the number of arrivals into the country (r=0.72, p=0.0004), and the health security index (r=0.51, p=0.0181). When we tried to normalize the cases and deaths by dividing by population, only COVID-19 containment and health index (r=0.23, p=0.03) and coronary artery disease death per 100,000 (r=0.46,p=0.0433) significantly correlated with case density (total number of cases/population for the country) and death density(total number of deaths/population for the country) respectively.

## Discussion

The low rate of COVID-19 cases and death in Africa represents a puzzle with different biological and social theories such as low COVID-19 testing capacity, substantial young population, few older adults, favorable climate, genetic admixture, infectious disease antibodies, and sound community health care systems [18]. We performed extensive data mining to aggregate data within the first six months of the pandemic (from the 22nd of January, 2020, to the 30th of June, 2020). We used secondary socio-economic data for the preceding years (2013-2019). In our study, Africa had the least number of cases and death despite being the second most populated continent. Similarly, our study population had a very low proportion of citizens above 65 with an impressive literacy rate. This literacy rate may have been significant in swift dissemination and response to preventive measures and strategies.

We assessed the basic health status and readiness of select African countries. The 2019 global health security index (GHSI) reported southern Africa to be the most prepared for a potential pandemic [5]. However, in our study, North Africa had the best health status based on assessed indices, and southern Africa was the most affected by COVID-19 (in terms of the number of cases, deaths, and GDP). However, they conducted more tests than other regions. East Africa was the least affected by COVID (cases and deaths), and West Africa had the lowest test rate. We also showed that the most effective preventive strategy was the use of face masks, particularly in reducing caseloads. The effectiveness of the face mask is as expected. Face masks are the most performing population-wide intervention to interrupt or reduce the spread of respiratory viruses [19]. Wang et al. reported that face masks were 79% effective in preventing transmission of COVID-19 provided it was strictly adhered to by all household members [20]. In a study of 18 African countries by the Partnership for Evidence-Based Response to COVID-19 (PERC), 85% of the respondents confirmed adherence to masks compared to other COVID-19 measures [21]. However, it is difficult to say if this effect is due to an isolated use of a face mask or its use in addition to other preventive measures.

We showed a correlation between the number of cases and deaths and the number of in-country arrivals, pandemic preparedness (health care security index), COVID-19 containment, and health index (not correlated with deaths). However, after normalizing cases and death with population, only COVID-19 containment and health index and coronary artery disease death per 100,000 were significantly correlated with case and death densities respectively. In Thailand, the number of tourists and their activities was significantly associated with COVID-19 cases [22]. Likewise, the relatively lower number of tourists to Africa (most of which are from African countries and very few from Asia) may have played a role in the low caseload on the continent. In addition, the rapid deployment of preventive measures may have contributed to the slower rise in cases and deaths at the beginning of the pandemic compared to western countries such as the USA and UK (Coronavirus chart: see how your country compares | Free to read | Financial Times {www.ft.com}). A perspective paper published by a group of early-career African scientists showed that 'the strengths of early initiated interventions may have contributed to modest and slower dynamics of COVID-19 in Sub-Saharan countries' like our finding [23]. This is despite the varying levels of adherence of Africans (low to high) [24-28].

We also evaluated the role of background health status on the COVID-19 case and death load. We assessed the correlation between hypertension, coronary artery disease death, Diabetes Mellitus, human immunodeficiency virus/ acquired immunodeficiency syndrome (HIV/AIDS), tuberculosis and kidney disease death rate per 100,000 and number of cases, number of deaths, COVID-19 cases, and death densities. We observed that only coronary artery disease rates correlated with COVID-19 death density. Our finding is corroborated by Liang et al. who revealed that coronary heart disease was associated with poor prognosis of COVID-19, mortality, severe/critical COVID-19, ICU admission, and disease progression [29].

Economically, only the North African countries experienced a GDP rise during the COVID pandemic. We cannot attribute the rise or fall in GDP strictly to the effect of COVID-19, but Martinho, in his study on the impact of COVID-19 on the GDP of OECD (The Organisation for Economic Co-operation and Development) countries, observed that the economic impacts from COVID‐19 were greater in countries with a higher incidence rate from the pandemic, in terms of total cases except Greece [30]. Maliszewka et al. had predicted that in a best-case scenario, the GDP in developing countries would fall by 2.5 percent and higher in an amplified pandemic scenario [31]. 

Our study did not specifically evaluate the effect of African genetic diversity and low rates of COVID-19 in Africa. People of African descent have been reported to have the lowest expression of angiotensin-converting enzyme 2 (ACE2) receptor levels, a receptor important in the cellular entry of severe acute respiratory syndrome coronavirus 2 (SARS-CoV-2) [32]. This low expression of ACE2 is hypothesized to offer genetic advantage like the novel resistance locus close to the cluster of genes that code for glycophorins (plasmodium falciparum erythrocyte invasion receptor), heterozygosity for sickle cell anemia, or resistance to *Plasmodium Vivax* infection due to the preponderance of Duffy blood group-negative individuals in Africa [33-35].

However, while Africans seemed relatively less susceptible, African Americans and black British were disproportionately affected by COVID-19 compared to other ethnic groups in America, with mortality at 97.9 per 100,000 [36,37]. This 'reduced susceptibility in Africans does not exist in the diasporic Africans, negating a transferable genetic component. However, even if there exists a 'reduced susceptibility' element', this effect appears to be masked by underlying socio-economic-disadvantages. Africans in the diaspora (both African immigrants and African Americans/Black British/Afro-Caribbean descendants) experience racism and discrimination, poverty, residential segregation, underlying health conditions, overcrowding, inconsistent access to health care, and work in essential fields limiting the ability to work from home [36,38], which interestingly also occurs in Africa. This puzzle is best answered by comparing African Americans or Black British to recent African immigrants. Unfortunately, no relevant COVID-19 data exists, but Turkson-Ocran et al. reported that risk factors for cardiovascular diseases (a predisposing factor for COVID-19 deaths) were lower in African immigrants than African Americans [39]. In addition, African immigrants are, in general, healthier than other immigrant and U.S born groups [40]. However, this may be more reflective of the health status of the immigrants (healthy citizens are more likely to immigrate) as opposed to the health status of Africans as a whole.

It is equally possible that Africa underreported COVID-19 cases and deaths due to inadequate testing capacity, underreporting and surveillance, and indeed African countries have performed the fewest COVID-19 tests compared to other countries. However, underreporting is unlikely to be solely responsible for the low cases and death rates. A disease as infectious as COVID-19 with an R naught (R0) between 1.50 and 6.68) [41] should have overwhelmed Africa's healthcare systems irrespective of the presence or absence of testing, as in other parts of the world [42,43]. Similarly, African countries would have reported a higher number of deaths and hospitalization, like in the Ebola outbreak between 2014 and 2015 [43]. In addition, there was no significant correlation between the number of tests performed and the COVID-19 cases and deaths among the 22 countries studied (r= -0.0027 to 0.265) in our study.

We also noted that our study population was relatively young. The median age of Africa (13 years) is at least 13 years less than the median age of the other continents. Likewise, the proportion of old adults (> 65 years) in Africa (3%) is at least 2.5 times less than in other continents, while the proportion of children (under 15 years) (41%) is almost 1.5 times higher than other continents. In addition, we found no correlation between the proportion of old people (age > 65 years) and COVID-19 cases or deaths. Lawal Y identified a positive correlation between the lower population mean age and COVID-19 mortality rate [44] in contrast with our study. Lawal Y had a wide variation in mean age with a range of (15.11 to 48.2 years). Furthermore, in contrast to the Lawal Y study, 1) we evaluated the population using a different metric; we used age > 65 while they used the mean, and 2) median age might have been more appropriate to use due to the skewed nature of population data. We used old age because COVID-19 mortality is highest among those aged 60 and above [45]. 

Fisman et al. suggested that the increased rates of COVID-19 in adults may be attributable to increased testing in this age group [46]. After correcting for increased testing in their study, they reported that adults aged 20-29 years were at higher risk for infection. Interestingly, in South Africa (on the 21st of April 2020), the age group with the highest number of cases were young people (30-34 years) [47,48], and death was highest among 60-64 years old, while in Zambia, it was 36-72 years (median age of 48). This age group is lower than Italy, Japan, and Spain's 60-69 year age group [35]. Hence, young African people are more likely to be infected with COVID-19, while older people are more likely to die due to COVID-19, contrary to Western expectations. Also, Oceania has the lowest burden of COVID-19 [49] but four times as many old adults as Africa and almost twice as fewer young people.

Likewise, the role of weather and climate is yet to be explicated. While air-drying capacity (ADC), which controls the fate of the virus-laden droplets, ultraviolet radiation (UV), and weather may explain up to 17% of the variation in COVID-19 growth rates, this percent is relatively small considering that 64% of the variation cannot be explained by environmental variables [50-52].

Strengths and limitations

The strength of this study is the enormous amount of data collected and the novelty of the research in the African context in an attempt to contribute to unraveling this puzzle. We were able to demonstrate relations between several interesting parameters which would be worth exploring further, while it would have been interesting to assess the strength of the relationships with regression analysis, we (the authors) collectively agree that the sample size (21 countries) would be insufficient for this analysis. But, this study has identified important factors such as arrivals into countries that should be explored in more detail. However, our study has some limitations which should be considered when interpreting our results. We studied just 21 countries out of 54 African countries due to time and resource constraints. Furthermore, It was difficult to account for the impact of increasing testing capacity over the study period (six months), however, for the African countries studied, there was minimal change in daily COVID-19 testing capacity at the initial stages, none large enough to perturb the relationship studied in this paper. We also observed that the findings in the COVID-19 case burden largely mirrored the death burden (a measure more resistant to the influence of change in testing capacities). It was difficult to account for the extra variability caused by differences in time of implementation of these preventive measures, some before the index case and some after, and the cumulative effect of implementing multiple preventive measures at once. In addition, we used data from multiple sources, potentially introducing variability, and performed correlation analysis to determine association and can thus make no inference about causation.

## Conclusions

The ongoing COVID-19 pandemic has significantly affected the African continent, although less than other continents. This effect also seems to extend economically with a general reduction in GDP in 2020 compared to pre-pandemic status, except for the North African countries. Africa had fewer COVID-19 cases and COVID-19 related deaths, and our data show that the rapidity and stringency of COVID-19 preventive measures and government policies, and the low level of tourism in Africa compared to other countries (i.e., low COVID-19 seeding rate) correlated with the case and death loads and may represent contributory factors to the African COVID-19 puzzle in addition to or asides from the most popular hypothesis which is underreporting of cases by African countries. We also discussed the unlikeliness of the African age demographics, low testing rate, and genetic resistance playing a significant role in explaining the low COVID-19 burden. Our study also showed a relationship between coronary artery disease and COVID-19 mortality. We hope this study helps to add to the existing body of knowledge on COVID-19 in Africa, highlight the potential relevance of the rapid adoption of pandemic preventive measures to flatten the curve, and identify new areas of research that can be further expanded on.

Future work

It would be interesting to explore the relationship between COVID-19 cases and COVID-19 related deaths and the rapidity and stringency of COVID-19 preventive measures and government policies, and the low level of tourism (i.e., low COVID-19 seeding across all African countries, or in fact globally). We are excited at future research that would deconvolute the cases and deaths by COVID-19 strains for example if these relationships exist with the strains that emerged later in 2020 and 2021.

## Appendices

The data and links to relevant sources for this paper can be accessed via this link  https://data.mendeley.com/datasets/xnrvphhy7m/1 all other data are available on reasonable request from the corresponding author.
